# Caspase-3 feedback loop enhances Bid-induced AIF/endoG and Bak activation in Bax and p53-independent manner

**DOI:** 10.1038/cddis.2015.276

**Published:** 2015-10-15

**Authors:** W Guo, Y Zhang, Z Ling, X Liu, X Zhao, Z Yuan, C Nie, Y Wei

**Affiliations:** 1 Department of Abdominal Oncology, State Key Laboratory of Biotherapy and Cancer Center, Collaborative Innovation Center for Biotherapy, West China Hospital, Sichuan University, 17# People's South Road, Chengdu, Chengdu 610041, PR China; 2 Departmant of Oncology, Guizhou People's Hospital, Guizhou 550002, PR China; 3 Departmant of Oncology, The Fourth People's Hospital of Sichuan province, Chengdu 610041, PR China

## Abstract

Chemoresistance in cancer has previously been attributed to gene mutations or deficiencies. Bax or p53 deficiency can lead to resistance to cancer drugs. We aimed to find an agent to overcome chemoresistance induced by Bax or p53 deficiency. Here, we used immunoblot, flow-cytometry analysis, gene interference, etc. to show that genistein, a major component of isoflavone that is known to have anti-tumor activities in a variety of models, induces Bax/p53-independent cell death in HCT116 Bax knockout (KO), HCT116 p53 KO, DU145 Bax KO, or DU145 p53 KO cells that express wild-type (WT) Bak. Bak knockdown (KD) only partially attenuated genistein-induced apoptosis. Further results indicated that the release of AIF and endoG also contributes to genistein-induced cell death, which is independent of Bak activation. Conversely, AIF and endoG knockdown had little effect on Bak activation. Knockdown of either AIF or endoG alone could not efficiently inhibit apoptosis in cells treated with genistein, whereas an AIF, endoG, and Bak triple knockdown almost completely attenuated apoptosis. Next, we found that the Akt-Bid pathway mediates Bak-induced caspase-dependent and AIF- and endoG-induced caspase-independent cell death. Moreover, downstream caspase-3 could enhance the release of AIF and endoG as well as Bak activation via a positive feedback loop. Taken together, our data elaborate the detailed mechanisms of genistein in Bax/p53-independent apoptosis and indicate that caspase-3-enhanced Bid activation initiates the cell death pathway. Our results also suggest that genistein may be an effective agent for overcoming chemoresistance in cancers with dysfunctional Bax and p53.

Mammalian cell death proceeds through a highly regulated program called apoptosis that is highly dependent on the mitochondria.^1^ Mitochondrial outer membrane (MOM) multiple apoptotic stresses permeabilize the MOM, resulting in the release of apoptogenic factors including cytochrome c, Smac, AIF, and endoG.^2, 3, 4^ Released cytochrome c activates Apaf-1, which assists in caspase activation. Then, activated caspases cleave cellular proteins and contribute to the morphological and biochemical changes associated with apoptosis. Bcl-2 family proteins control a crucial apoptosis checkpoint in the mitochondria.^2, 5, 6, 7^ Multidomain proapoptotic Bax and Bak are essential effectors responsible for the permeabilization of the MOM, whereas anti-apoptotic Bcl-2, Bcl-xL, and Mcl-1 preserve mitochondrial integrity and prevent cytochrome c efflux triggered by apoptotic stimuli. The third Bcl-2 subfamily of proteins, BH3-only molecules (BH3s), promotes apoptosis by either activating Bax/Bak or inactivating Bcl-2/Bcl-xL/Mcl-1.^8, 9, 10, 11, 12^ Upon apoptosis, the ‘activator' BH3s, including truncated Bid (tBid), Bim, and Puma, activate Bax and Bak to mediate cytochrome c efflux, leading to caspase activation.^8, 11, 12^ Conversely, antiapoptotic Bcl-2, Bcl-xL, and Mcl-1 sequester activator BH3s into inert complexes, which prevents Bax/Bak activation.^8, 9^ Although it has been proposed that Bax and Bak activation occurs by default as long as all of the anti-apoptotic Bcl-2 proteins are neutralized by BH3s,^13^ liposome studies clearly recapitulate the direct activation model in which tBid or BH3 domain peptides derived from Bid or Bim induce Bax or Bak oligomerization and membrane permeabilization.^12, 14, 15^

Numerous studies have demonstrated a critical role for Bax in determining tumor cell sensitivity to drug induction and in tumor development. Bax has been reported to be mutated in colon^16, 17^ and prostate cancers,^18, 19^ contributing to tumor cell survival and promoting clonal expansion. Bax has been shown to restrain tumorigenesis^20^ and is necessary for tBid-induced cancer cell apoptosis.^21^ Loss of Bax has been reported to promote tumor development in animal models.^22^ Bax knockout (KO) renders HCT116 cells resistant to a series of apoptosis inducers.^23, 24, 25^ p53 has been reported to be a tumor suppressor,^26^ and its mutant can cause chemoresistance in cancer cells.^27, 28, 29^ Moreover, p53 is often inactivated in solid tumors via deletions or point mutations.^30, 31^ Thus, it is necessary to find an efficient approach or agent to overcome chemoresistance caused by Bax and/or p53 mutants.

Few studies have focused on the role of Bak in tumor cell apoptosis and cancer development. Bak mutations have only been shown in gastric and colon cancer cells.^32^ Some studies have revealed that Bak is a determinant of cancer cell apoptosis.^33, 34^ Some studies have even demonstrated that Bak renders Bax KO cells sensitive to drug induction.^33, 35^ In this study, we are the first group to show that tBid induces Bak activation and the release of AIF and endoG in colon cancer cells, which causes cellular apoptosis independent of Bax/p53. We also found that caspase-3 is activated in apoptosis. Interestingly, downstream caspase-3 can strengthen Bak activation and the release of AIF and endoG during apoptosis via a feedback loop. Furthermore, we reveal that Akt upregulates apoptosis progression. These results will help us to better understand the function of mitochondrial apoptotic protein members in apoptosis and cancer therapies. Furthermore, our experiments may provide a theoretical basis for overcoming chemoresistance in cancer cells.

## Results

### Genistein induces Bax and p53-independent apoptosis in cancer cells

**Figure 1:**
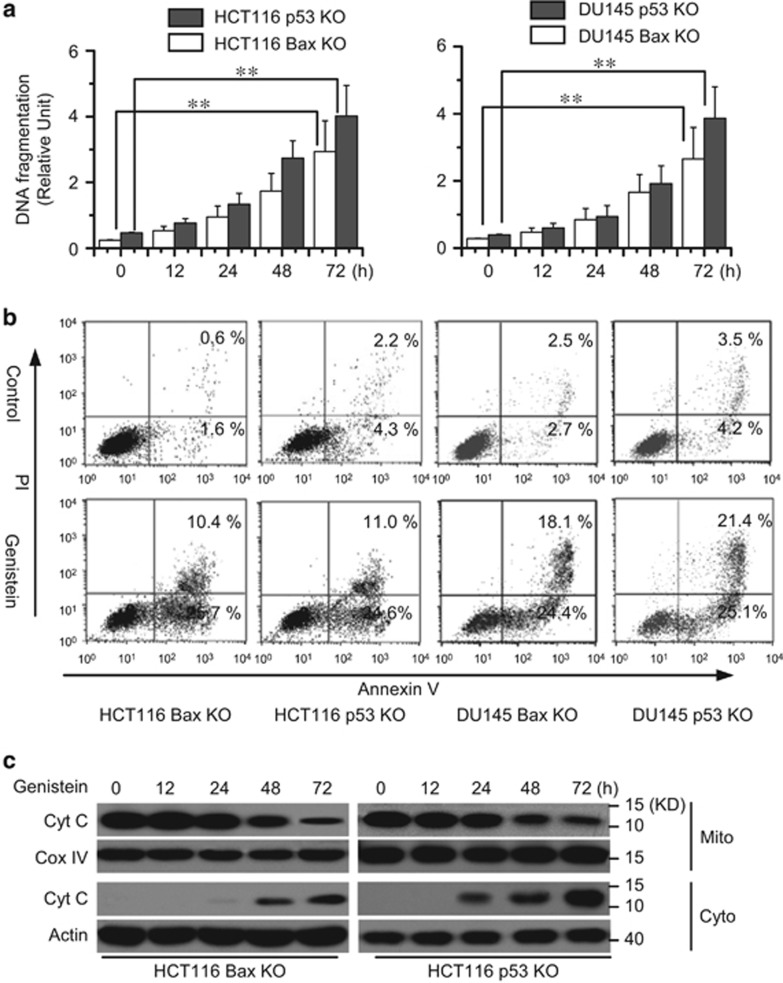
Genistein induces cell apoptosis in Bax and p53-independent manner. (**a**) Analysis of cell apoptosis treated with genistein. Cells were treated with genistein (30 *μ*M) for different periods of time and then collected to examine apoptosis. Cell apoptosis was quantitatively detected by a cell death ELISA kit as described in Materials and methods. Graphs showing results of quantitative analyses (*n*=3, mean±S.D., **P<0.01). (**b**) Detection of cell apoptosis with flow cytometry. Cells were treated with genistein (30 *μ*M) for 72 h, and then collected for Annexin V and PI double staining with flow cytometry. Apoptotic cells were assessed with Annexin V positive and PI negative. (**c**) Cell were treated with genistein (30 *μ*M) for different periods of time, and subjected to subcellular fractionation. The cytosolic or mitochondrial fractions were immunoblotted for Cyt c detection. *β*-Actin and Cox IV were used as a protein loading control. Representative results of three experiments with consistent results are shown

**Figure 2:**
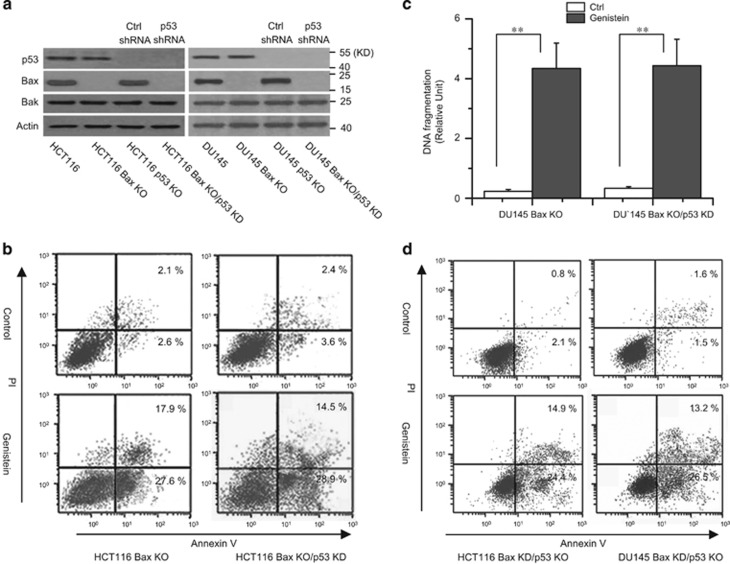
The effect of p53 in Bax KO cells. (**a**) Cells were stably transfected with Ctrl or p53 shRNA and transfected cells were immunoblotted for p53, Bax, Bak detection. *β*-Actin was used as a protein loading control. (**b**) Cells were treated with genistein (30 *μ*M) for 72 h. Cell apoptosis was detected with Annexin V/PI staining. (**c**) Cells were treated with genistein (30 *μ*M) for 72 h. Cell death was detected with ELISA. Graphs showing results of quantitative analyses (*n*=3, mean±S.D., **P<0.01). (**d**) HCT116 ad DU145 p53 KO cells were transiently transfected with Bax siRNA for 48 h, and then cells were treated with with genistein for 72 h. Cell apoptosis was detected with Annexin V/PI staining. Representative results of three experiments with consistent results are shown

We first determined the apoptotic effects of genistein in HCT116 Bax KO and p53 KO cells. We treated the cells with genistein at the indicated time, and apoptosis was assessed by a DNA fragmentation ELISA. As depicted in Figure 1a, genistein efficiently induced cell death in HCT116 Bax KO, p53 KO, DU145 KO, and p53 KO cells. Flow-cytometry analysis with Annexin V/PI staining also revealed that genistein could induce apoptosis in these cell lines (Figure 1b). Moreover, our data demonstrated that genistein effectively induced Cyt c release from the mitochondria to the cytosol (Figure 1c). Next, we stably transfected p53 shRNAs into HCT116 Bax KO or DU145 Bax KO cells to make p53 knockdown cell lines (Figure 2a). Both flow-cytometry analysis (Figure 2b) and ELISA (Figure 2c) revealed that p53 knockdown had little effect on cell apoptosis by genistein. Meanwhile, we transiently transfected Bax siRNA (si Bax) into HCT116 p53 KO or DU145 p53 KO cells to make Bax knockdown cell lines (Supplementary Figure 1A). Flow-cytometry analysis demonstrated that Bax deficiency also had little effect on cell death in p53 KO cells (Figure 2d). These results indicate that genistein caused apoptosis independent of Bax and p53.

### Bak activation is important for genistein-induced apoptosis

**Figure 3:**
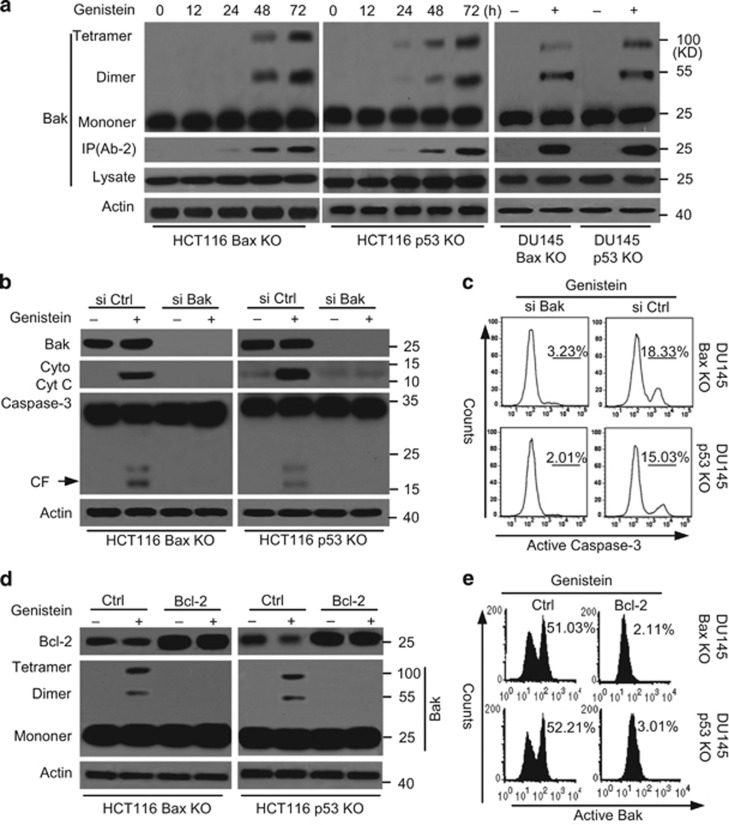

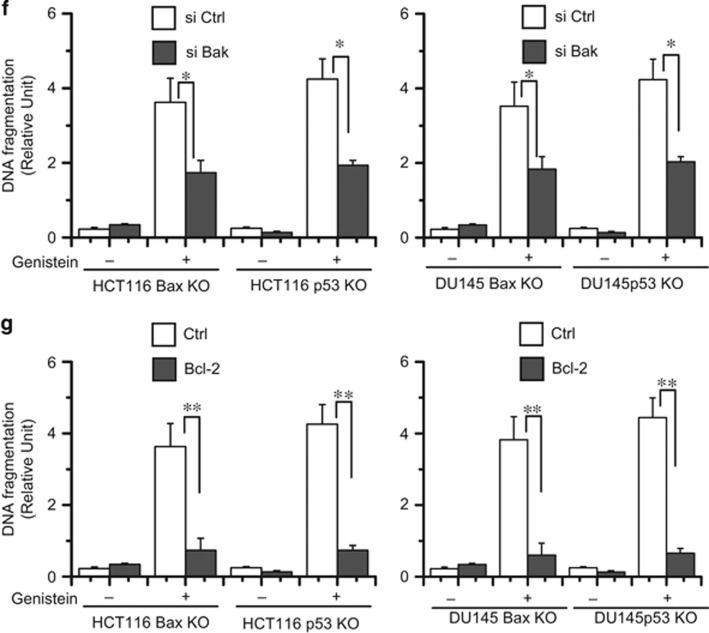
Bak is required for genistein-induced apoptosis. (**a**) Time-dependent analysis of Bak activation in cells treated with genistein (30 *μ*M) at the indicated time. Cells were treated with genistein (30 *μ*M), and then collected for detection. The oligomerization of Bak was assessed by cross-linking with Bismaleimidohexane (BMH) as described in Materials and methods. Treated cells were lysed in lysis buffer, and Bak was detected by western blotting with anti-Bak antibody (Sigma). For the conformation of Bak detection, treated cells were lysed in Chaps buffer and subjected to immunoprecipitation with anti-Bak Ab-2 antibody from Calbiochem. *β*-Actin was used as a protein loading control. (**b**) Cells were transfected with Ctrl or Bak siRNA for 48 h and treated with genistein (30 *μ*M) for 48 h. One portion of treated cells was subjected to subcellular fraction and detection of Cyt c release. The other portion of treated cells was used to detect Bak expression and caspase-3 cleavage. (**c**) As described in (**b**), cells were permeabilized, fixed, and stained for active caspase-3 and analyzed by flow cytometry. (**d**) Cells were transfected with Ctrl or Bcl-2 vector for 48 h and treated with genistein (30 *μ*M) for 48 h. Treated cells were collected for western blot analysis. (**e**) Cells were permeabilized, fixed, and stained for active Bak with Ab-2 antibody and analyzed by flow cytometry. (**f**) Cells were transfected with Ctrl or Bak siRNA for 48 h and treated with genistein (30 *μ*M) for 72 h. Cell death was detected with ELSIA. Graphs showing results of quantitative analyses (*n*=3, mean±S.D., *P<0.05). (**g**) Cells were transfected with Ctrl or Bcl-2 vector for 48 h and treated with genistein (30 *μ*M) for 72 h. Cell death was detected with ELSIA. Graphs showing results of quantitative analyses (*n*=3, mean±S.D., **P<0.01). All data are representative of three independent experiments

Because Bak contributes to Bax-independent cell death,^33, 36^ we speculated that Bak could mediate genistein-induced Bax/p53-independent cell death. Our experiments revealed that genistein induced Bak oligomerization and a conformational change in p53 or Bax KO cells (Figure 3a). Meanwhile, we still detected Bak oligomerization in Bax KD/p53 KO or Bax KO/p53 KD cells. Genistein treatment was still sufficient to induce Bak oligomerization in Bax KD/p53 KO or Bax KO/p53 KD cancer cells (Supplementary Figure 1B).

To further investigate the contribution of Bak to cell apoptosis, we transfected Bak siRNAs (si Bak) into HCT116 Bax KO or p53 KO cells to knock down Bak expression. Western blot analysis confirmed the lack of Bak expression in cancer cells (Figure 3b). As illustrated in Figures 3b and c, Bak siRNAs efficiently decreased Cyt c release and caspase-3 activation. Meanwhile, Bak knockdown also decreased cell death in cancer cells treated with genistein (Figure 3f). Further experiments revealed that Bcl-2 overexpression obviously inhibited Bak oligomerization and the conformational change as well as apoptosis by genistein (Figures 3d, e, and g). These results suggest that Bak has an important role in genistein-induced Bax/p53-independent cell apoptosis.

### The release of AIF and endoG from the mitochondria to the cytosol mediates apoptosis

**Figure 4:**
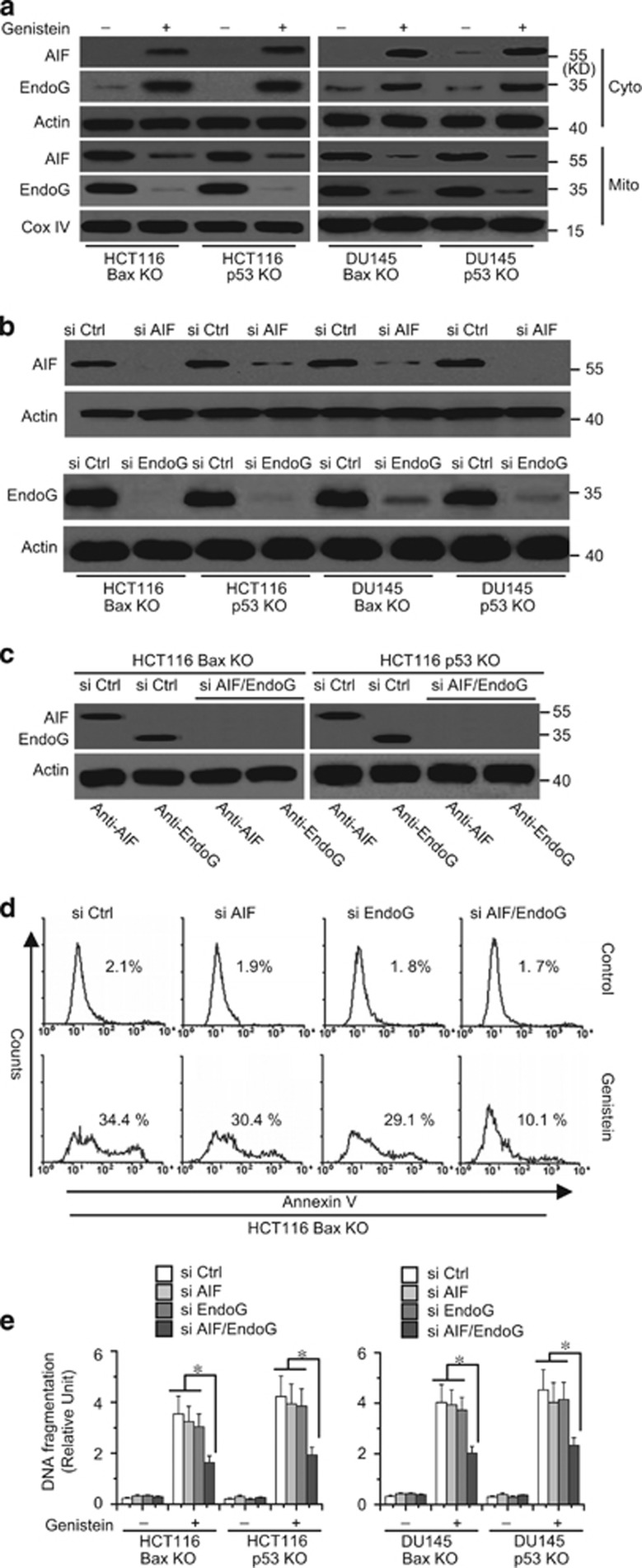
AIF and endoG has an important role in apoptosis. (**a**) Cells were treated with genistein (30 *μ*M) for 72 h, and subjected to subcellular fractionation. The cytosolic or mitochondrial fractions were immunoblotted for AIF and endoG detection. *β*-Actin and Cox IV were used as a protein loading control. (**b**) Cells were transfected with AIF or endoG siRNA for 48 h, and then transfected cells were immunoblotted for AIF and endoG detection. (**c**) HCT116 Bax KO or p53 KO cells were transfected with AIF and siRNA for 48 h, and then transfected cells were immunoblotted for AIF and endoG detection. β-*A*ctin was used as a protein loading control. (**d**) HCT116 Bax KO cells were transfected with AIF, endoG siRNA, or double siRNA for 48 h, and then cells were treated with genistein for 72 h. Collected cells were stained with Annexin V for apoptosis analysis. (**e**) Cells were transfected with AIF, endoG siRNA, or double siRNA for 48 h, and then treated with genistein for 72 h. Cell apoptosis was quantitatively detected by a cell death ELISA kit as described in Materials and methods. Graphs showing results of quantitative analyses (*n*=3, mean±S.D., *P<0.05). Representative results of three experiments with consistent results are shown

Our experiments also found that genistein induced the release of AIF and endoG in Bax or p53 KO cancer cells (Figure 4a). AIF and endoG are important factors for mitochondrial apoptosis and can mediate caspase-dependent and -independent cell death.^3, 37^ We then used AIF siRNAs (si AIF), endoG siRNAs (si endoG), or both siRNAs (si AIF/endoG) to decrease AIF and endoG expression in cancer cells (Figures 4b and c). We found that knockdown of either AIF or endoG alone could not efficiently decrease apoptosis in cells treated with genistein. However, the AIF and endoG double knockdown substantially decreased apoptosis (Figures 4d and e). These results indicate that the release of mitochondrial AIF and endoG is necessary for genistein-induced Bax/p53-independent cell apoptosis.

### Bid causes Bak activation and the release of AIF and endoG during apoptosis

**Figure 5:**
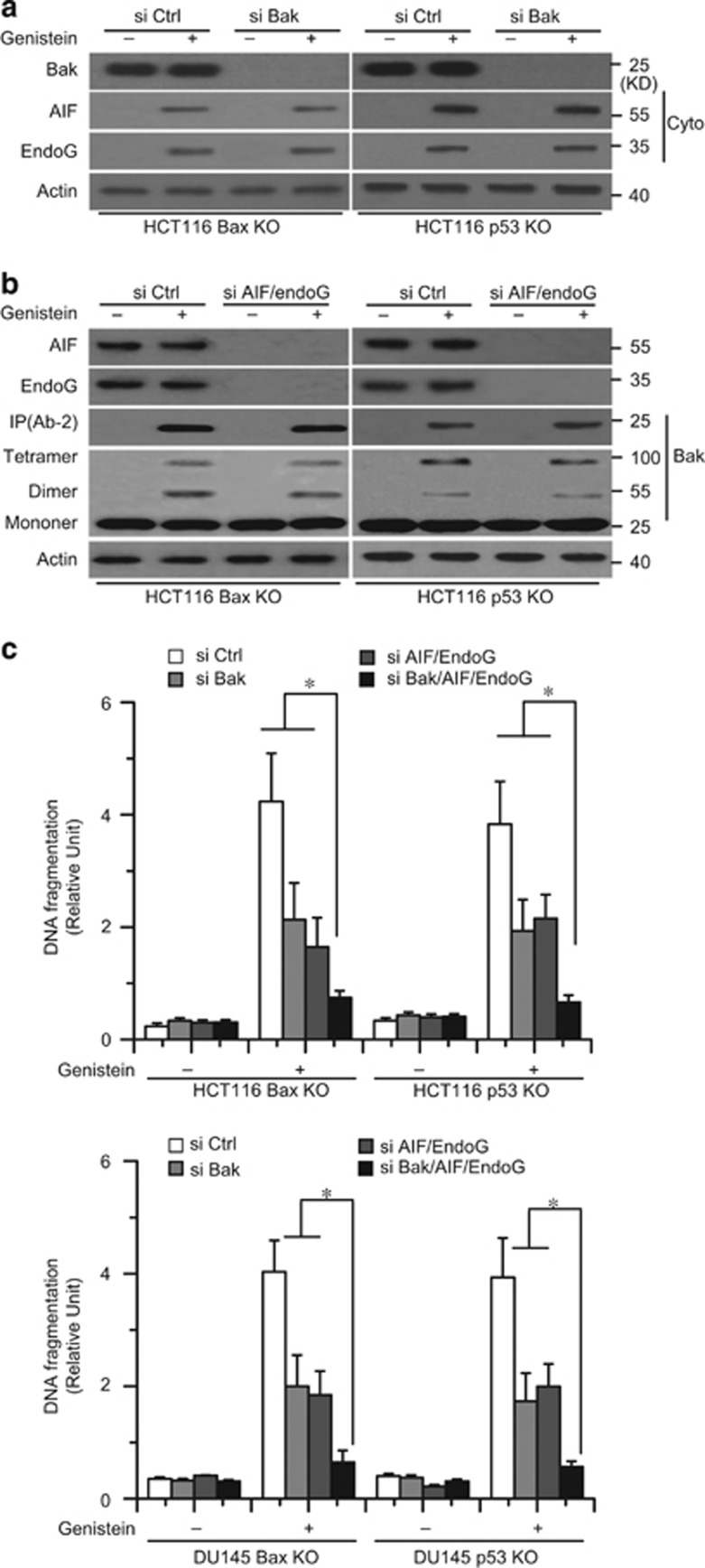
The interaction of Bak or AIF and endoG knockdown. (**a**) Cells were transfected with Ctrl or Bak siRNA for 48 h and treated with genistein (30 *μ*M) for 48 h. Treated cells were used to detect Bak, AIF, and endoG expression. (**b**) Cells were transfected with Ctrl or double AIF or endoG siRNA for 48 h and treated with genistein (30 *μ*M) for 48 h. Treated cells were used to detect Bak, AIF, and endoG expression. *β*-Actin was used as a protein loading control. (**c**) Cells were treated with single AIF, endoG, Bak, Ctrl siRNA, double AIF/endoG, or triple AIF/endoG/Bak siRNA for 48 h, and then treated with genistein for 72 h. Cell apoptosis was quantitatively detected by a cell death ELISA kit as described in Materials and methods. Graphs showing results of quantitative analyses (*n*=3, mean±S.D., *P<0.05)

Our study revealed that Bak could induce the release of Cyt c from the mitochondria to the cytosol; thus, we detected whether Bak induced the release of AIF and endoG during apoptosis. We knocked down the expression of Bak with siRNAs in cancer cells. We found that Bak knockdown had little effect on the release of AIF and endoG (Figure 5a). Similarly, AIF and endoG knockdown had little effect on Bak activation (Figure 5b). These results indicate that Bak activation and the release of AIF and endoG are likely parallel apoptotic events. Indeed, we found that a Bak, AIF, and endoG triple knockdown almost completely inhibited apoptosis in cells treated with genistein (Figure 5c). We speculated that the other factors can cause Bak activation as well as the release of AIF and endoG during apoptosis.

**Figure 6:**
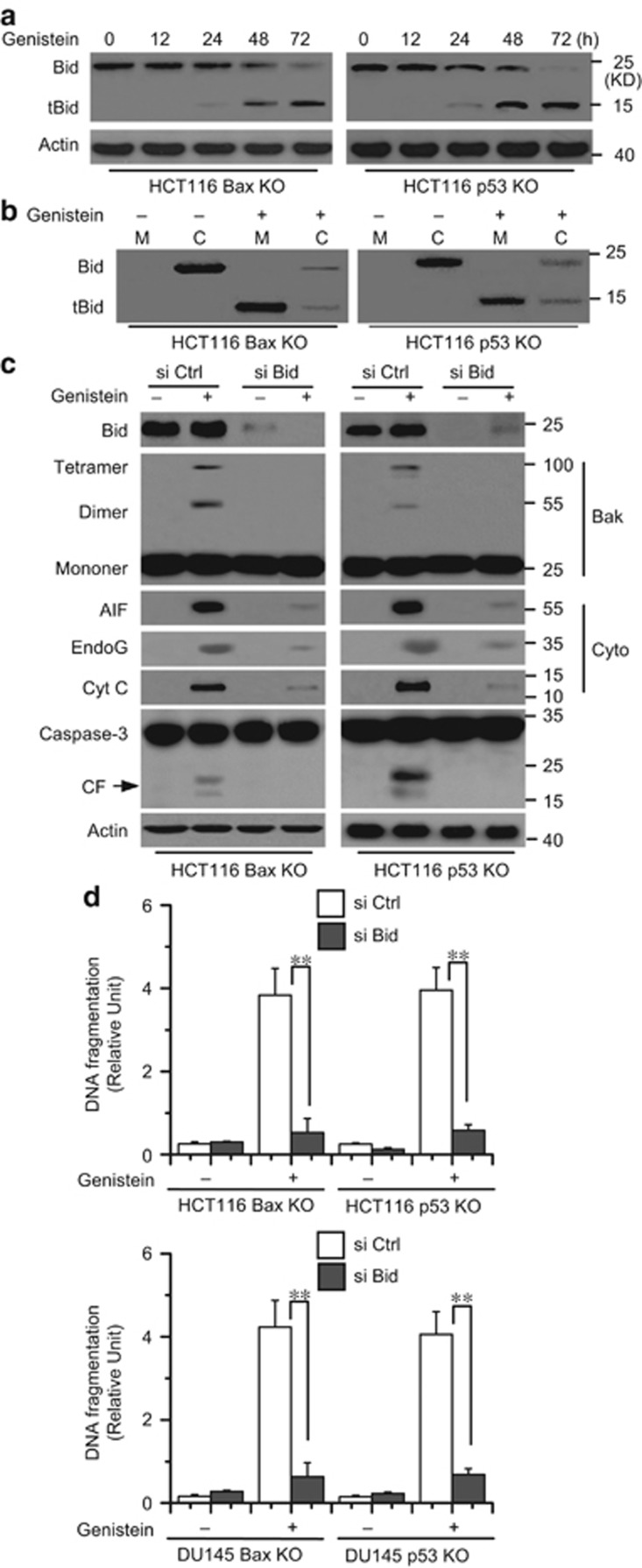
Bid activation mediates AIF, endoG release, and Bak activation. (**a**) Cell were treated with genistein at indicated time and collected with western blot analysis. *β*-Actin was used as a protein loading control. (**b**) Cells were treated with genistein for 72 h and subjected to subcellular fractionation. The cytosolic (C) or mitochondrial (M) fractions were immunoblotted for Bid and tBid detection. (**c**) Cells were transfected with Bid and Ctrl siRNA for 48 h, and then treated with genistein for 72 h. One portion of cells was subjected to subcellular fraction to detect the release of AIF, endoG, and Cyt c. The other portion of cells was collected to detect Bak and caspase activation. *β*-Actin was used as a protein loading control. (**d**) Cells were transfected with si Ctrl or si Bid for 48 h and treated with genistein (30 *μ*M) for 72 h. Cell death was detected with ELSIA. Graphs showing results of quantitative analyses (*n*=3, mean±S.D., **P<0.01). Representative results of three experiments with consistent results are shown

Previous studies have demonstrated that activated Bid can mediate Bak activation and the release of AIF and endoG.^38, 39, 40^ We then detected whether Bid contributes to genistein-induced cell apoptosis. We first detected Bid expression and activation. Our data confirmed that Bid was cleaved into tBid (Figure 6a), which is the active truncated form of Bid,^38^ during the time course of genistein treatment. We also found that tBid translocated into the mitochondria from the cytosol (Figure 6b), which could induce the release of mitochondrial proteins.^41^ We then knocked down the expression of Bid with siRNAs. Our data revealed that Bid expression was inhibited by siRNA treatment (Figure 6c). Moreover, Bid knockdown affected Bak oligomerization; the release of Cyt c, AIF, and endoG; and caspase-3 cleavage in Bax or p53 KO cells (Figure 6c). Meanwhile, Bid knockdown also substantially decreased apoptosis in Bax or p53 KO cells treated with genistein (Figure 6d). We also transiently transfected Bid siRNAs into Bax KD/p53 KO or Bax KO/p53 KD cells. We found that Bid knockdown is sufficient to attenuate Bak oligomerization; the release of Cyt c, AIF, and endoG; and caspase-3 cleavage in cancer cells (Supplementary Figures 1C and D). These results demonstrated that Bid could upregulate Bak activation; the release of AIF and endoG; and subsequently apoptosis.

### Akt inactivation mediates Bid-induced cell apoptosis

**Figure 7:**
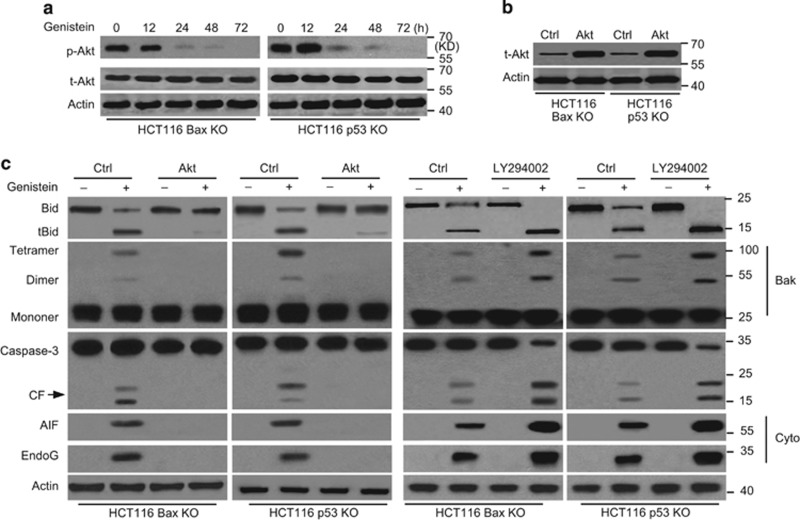
Inactivated Akt upregulates Bid-induced Bak activation, the release of AIF and endoG. (**a**) Cells were treated with genistein at indicated time and immunoblotted for phosphor-Akt (p-Akt) and total Akt (t-Akt) detection. *β*-Actin was used as a protein loading control. (**b**) Cells were transfected with Akt1 or Ctrl vector for 48 h and then transfected cells were immunoblotted for t-Akt detection. (**c**) Cells were transfected with Akt1 or Ctrl vector for 48 h, and then treated with genistein for 72 h or cells were treated with genistein and/or 25 *μ*M LY294002 for 72 h. Treated cells were immunoblotted for detection. *β*-Actin was used as a protein loading control. All data are representative of three independent experiments

Previous studies have revealed that Akt activation could inhibit tBid appearance and Bid-induced apoptosis.^42, 43^ Moreover, a previous study showed that genistein induced apoptosis through the Akt pathway in anaplastic large-cell lymphoma.^44^ Thus, we speculated that Akt is the upstream apoptotic regulator in our experiments. We first detected phosphorylated Akt (p-Akt) and total Akt (t-Akt) expression. We found that p-Akt was decreased during the time course of genistein treatment. Meanwhile, t-Akt had little change after treatment (Figure 7a). We then further detected the effects of Akt on apoptosis. We transfected a constitutively active Akt1 construct into cancer cells and found that Akt1 overexpression increased t-Akt in cells (Figure 7b). Moreover, Akt1 overexpression obviously inhibited the appearance of tBid, Bak oligomerization, caspase-3 cleavage, and the release of AIF and endoG (Figure 7c). Our experiments also revealed that LY294002, a PI3K/Akt pathway inhibitor that inactivates Akt, efficiently increased Bid activation, Bak oligomerization, caspase-3 cleavage, and the release of AIF and endoG (Figure 7c). These results indicate that Akt inactivation mediates Bid-induced downstream apoptotic events instigated by genistein.

### Caspase-3 enhances Bid-induced Bak activation and the release of AIF and endoG

**Figure 8:**
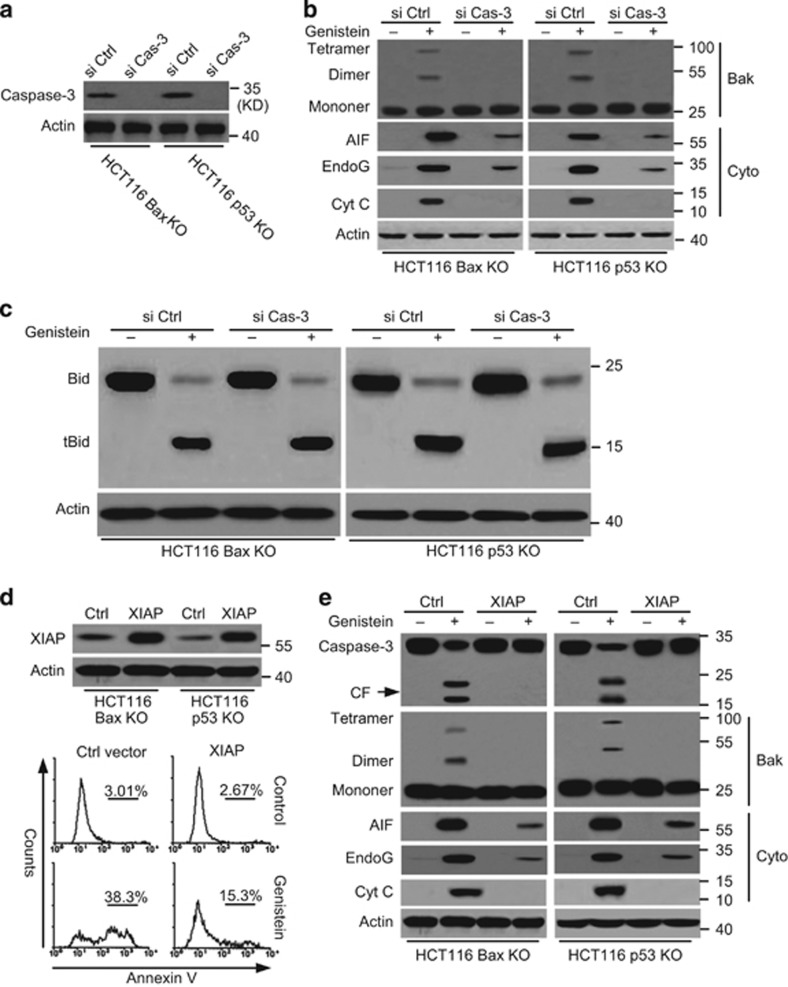
Caspase-3 feedback regulates AIF, endoG release, and Bak activation. (**a**) Cells were transfected with si Ctrl or si Caspase-3 for 48 h and then transfected cells were immunoblotted for caspase-3 detection. (**b**) Cells were transfected with si Ctrl or si Caspase-3 for 48 h and treated with genistein for 72 h. Treated cells were detected for Bak oligomerization, AIF, and endoG release, etc. (**c**) Cells were transfected with si Ctrl or Caspase-3 siRNA for 48 h and treated with genistein for 72 h. Treated cells were detected for Bid cleavage. β-Actin was used as a protein loading control. (**d**) *Up*, Cells were transfected with XIAP or Ctrl vector for 48 h and lysed for immunoblotted detection. *Down*, HCT116 Bax KO cells were transfected with XIAP or Ctrl vector for 48 h and treated with genistein for 72 h. Treated cells were detected cell apoptosis with flow cytometry. (**e**) Cells were transfected with XIAP or Ctrl vector for 48 h and treated with genistein for 72 h. Treated cells were detected for Bax oligomerization, AIF, and endoG release, etc. *β*-Actin was used as a protein loading control. Representative results of three experiments with consistent results are shown

Previous reports have revealed that downstream apoptotic factors, such as Smac and caspase-9, could mediate upstream apoptotic events through positive feedback.^2, 5, 45^ Thus, we detected whether caspase-3 could enhance Bak activation. We first knocked down caspase-3 with siRNAs (si Cas-3). We found that caspase-3 expression was inhibited by siRNAs in cancer cells (Figure 8a). Our data indeed revealed that caspase-3 knockdown decreased Cyt c release and Bak oligomerization (Figure 8b). However, interestingly, caspase-3 knockdown also obviously decreased the release of AIF and endoG (Figure 8b). Moreover, caspase-3 knockdown had little effect on Bid activation (Figure 8c). These results suggest that downstream caspase-3 could enhance Bid-induced subsequent apoptotic events, but did not affect Bid activation.

To further examine the effect of caspase-3 during apoptosis, we transfected an XIAP construct into cancer cells to antagonize caspase-3 activation. The cleavage of caspase-3 could be blocked by XIAP overexpression. XIAP overexpression could also decrease apoptosis.^46^ Our data revealed that the XIAP construct increased XIAP overexpression and decreased apoptosis in cells treated with genistein (Figure 8d). XIAP overexpression inhibited caspase-3 cleavage, Bak oligomerization, and the release of Cyt c. XIAP overexpression also decreased the release of AIF and endoG from the mitochondria (Figure 8e). These results further revealed that downstream caspase-3 could enhance Bid-induced apoptosis.

## Discussion

Genistein, a naturally occurring isoflavone and prominent isoflavonoid found in soy products, is of interest because of its potent chemotherapeutic activities, including its ability to inhibit cell growth and induce apoptosis in a wide variety of cultured cancer cells.^44, 47, 48, 49, 50, 51^ Moreover, genistein has chemopreventive effects in several human malignancies, including cancers of the breast, colon, and prostate.^49, 52^ Previous studies have revealed that genistein induces apoptosis in breast cancer cells by upregulating pro-apoptotic Bcl-2 proteins, such as Bax or Bak, and downregulating anti-apoptotic factors, such as Bcl-xL or Bcl-2.^48, 53^ Some studies have also revealed that genistein involves the Akt signaling pathway in apoptosis.^44, 47^ However, the exact function of genistein on cancer chemoresistance is still unknown.

In the present study, we first demonstrated that genistein can induce apoptosis independent of Bax and p53. Previous studies have demonstrated that genistein induced apoptosis independent of p53.^53^ We focused on genistein and found out that genistein can induce cell death in the absence of Bax and p53. We found that Bak compensates for the loss of Bax in cancer cell apoptosis. However, Bak knockdown only partially inhibited apoptosis in cells treated with genistein. These results suggest that Bak may not be the sole decisive factor for genistein-induced apoptosis.

Mitochondria respond to multiple death stimuli including those involving pro-apoptotic Bcl-2 family proteins, such as Bax and Bak, which induce mitochondrial membrane permeabilization and cause the release of apoptotic molecules.^2, 6, 33, 38^ Therefore, we speculated that Bak could mediate the release of some mitochondrial apoptotic factors, such as Cyt c, AIF, and endoG. Indeed, our data revealed that the release of AIF and endoG from that mitochondria was an important part of genistein-induced cell death. It has been reported that AIF and endoG translocate to the nucleus to trigger caspase-independent cell death.^37^ Our data also revealed that Bak mediated the release of Cyt c. Bak knockdown efficiently inhibited the release of Cyt c. However, interestingly, Bak knockdown had little effect on the release of AIF and endoG. Conversely, an AIF and endoG double knockdown did not affect Bak activation. These results lead us to believe that another upstream factor mediates genistein-induced apoptosis.

Previous studies have also revealed that the Akt signaling pathway contributes to genistein-induced apoptosis^44, 47^ Our data further demonstrated that the Akt-Bid signaling pathway upregulates Bak activation and the release of AIF and endoG. Further experiments revealed that downstream caspase-3 could enhance Bak activation and the release of AIF and endoG. Arnoult *et al.*^3^ revealed that mitochondrial 'release of AIF and endoG required caspase activation. Our study also confirmed this conclusion. Moreover, we also proved that Bak did not contribute to the release of AIF and endoG. Arnoult *et al.* also found that tBid could induce the release of Cyt c, but not AIF and endoG. However, our research revealed that tBid could induce the release of Cyt c, AIF, and endoG, as described previously.^39, 54^ Furthermore, we also found that caspase-3 inhibition decreased the release of mitochondrial apoptotic factors, whereas previous studies have shown that caspase inhibitors prevent the mitochondrial release of AIF and endoG, but not Cyt c. This result further suggests that we have found a different mechanism underlying the regulation of Bak activation and the release of AIF and endoG.

**Figure 9:**
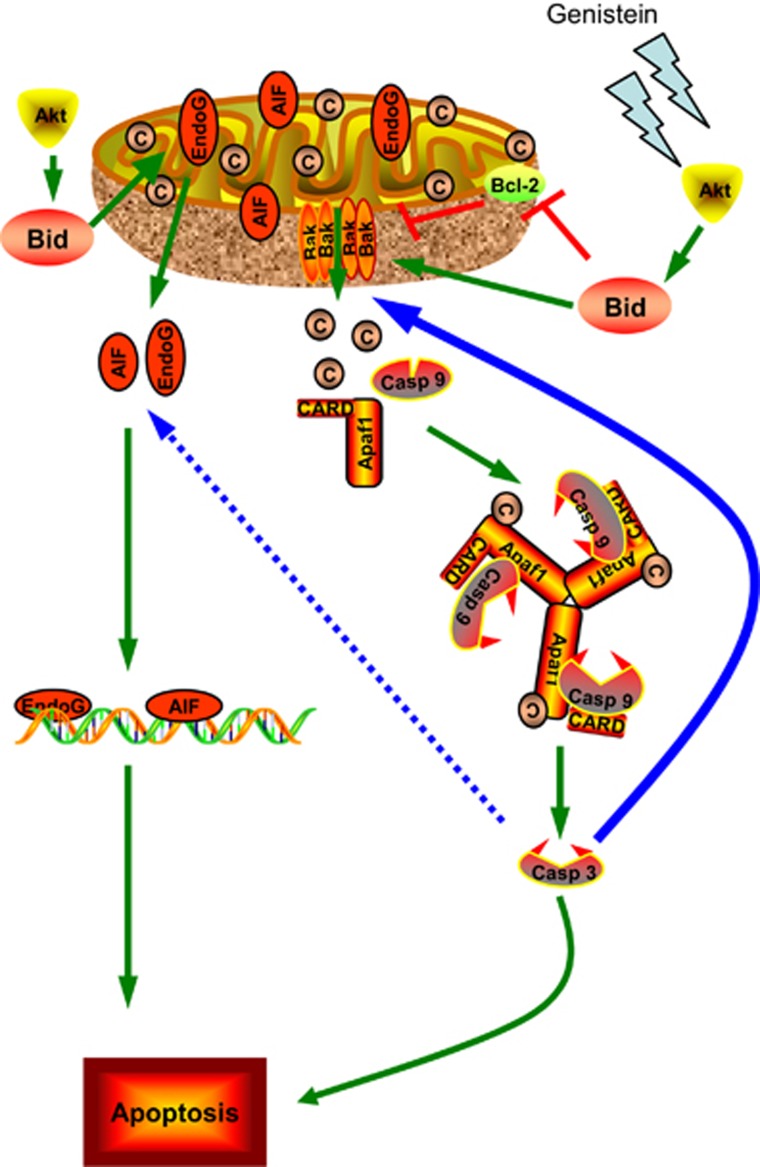
A diagram of signaling pathway for genistein-induced apoptosis in Bax and p53-independent manner. Genistein first induced Akt dephosphorylation. p-Akt decrease activated Bid to tBid. Bid cleavage induced the release of AIF and endoG as well as Bak activation. Activated Bak triggered caspase-dependent apoptosis, while AIF and endoG release initiated caspase-independent cell death. Activated caspase-3 mediated two positive feedback loops to enhance apoptosis. On one hand, caspase-3 feedback enhances Bak activation (the blue solid line); on the other hand, caspase-3 probably strengthened the release of AIF and endoG (the blue dotted line)

In conclusion, we explored, for the first time, the detailed molecular mechanisms of Bid-induced apoptosis after genistein treatment and demonstrate that caspase-3 could enhance genistein-induced apoptosis. Our findings explain how genistein induces apoptosis in Bax- and p53-deficient cancer cells. The Akt-Bid pathway initiates Bak activation and downstream caspase processing. Meanwhile, the Akt-Bid pathway also causes the release of AIF and endoG, which induces caspase-independent cell death. Downstream caspase-3 reinforces Bak activation and the release of AIF and endoG through positive amplification loops (Figure 9). These positive regulatory feedback loops sensitize cancer cells to treatment with genistein. Genistein seems to have broader implications, even in a clinical perspective.

## Materials and Methods

### Materials

Genistein, LY294002, Annexin V, and PI were obtained from Sigma (St. Louis, MO, USA). Bak (B5897) and actin (clone AC-74, A5316) antibodies were also from Sigma. Bismaleimidohexane (BMH) was obtained from Pierce (Rockford, IL, USA). Prestained marker (SM0671) was from Fermentas (Vilnius, Lithuania). Bak (Ab-2) was purchased from Calbiochem (San Diego, CA, USA). p53 (clone 7F5, #2527), phospho-Akt (Ser 473) (clone 587F11, #4051), Akt (#9272), caspase-3 (clone 8G10, #9665), Bid (#2002), XIAP (#2042), AIF (#4642), and Cox IV (#4844) were purchased from Cell Signaling (Beverly, MA, USA). EndoG (ab9647) antibody was from Abcam (Cambridge, UK). Cyt c (sc-13156), Bcl-2 (sc-7382), and Bax N-20 (sc-493) antibodies were from Santa Cruz (Santa Cruz, CA, USA).

### Gene silencing with small interfering rnas and plasmids

Small interfering RNA (siRNA) oligonucleotides were purchased from Dharmacon (Lafayette, CO, USA) with sequences targeting Bax (5′-AACUGAUCAGAACCAUCAUGG-3′), Bak (5′-AACCGACGCUAUGACUCAGAG-3′), Bid (5′-AAGAAGACAUCAUCCGGAAUA-3′) AIF (5′-GGCUACGUCCAGGAGCGCACC-3′), endoG (5′-AAGAGCCGCGAGUCGUACGUG-3′), caspase-3 (5′-UGAGGUAGCUUCAUAGUGGTT-3′), and p53 (5′-CGGCAUGAACCGGAGGCCCAU-3′). For p53 shRNA construction, the siRNA was cloned into the pSilencer 2.1-U6 hygro plasmid. The constitutively active Akt1 construct HA-PKB-T308D/S473D was obtained as previously described.^5, 55^

### Cell culture and transfection

DU145 and HCT116 cells were obtained from the American Type Culture Collection. DU145 and HCT116 cells were incubated in DMEM supplemented with 10% FBS and penicillin-streptomycin. HCT116 Bax KO^6^ cells were the gift from Quan Chen (Chinese Academy of Sciences, Beijing, China). DU145 Bax KO, DU 145 p53 KO, and HCT116 p53 KO were obtained from Dean G. Tang (the University of Texas MD Anderson Cancer Center, Science Park-Research Division, Smithville, TX, USA).

For siRNA or shRNA transfection, cells were seeded on 6-well plates and then transfected with the appropriate plasmid DNA or siRNA using the manufacturers' protocols. Typically, cells were seeded on coverslips in the 6-well plates, and then 1 *μ*g of plasmid DNA or 100 nM siRNA and 4 *μ*l of DMRIE-C reagent (Invitrogen, Carlsbad, CA, USA) were used per coverslip. The cells were incubated for 4 h in the transfection mixture, which was then replaced with fresh culture medium. For stable transfection, cells were transfected with the constructs as previously described.^2, 6^ Positive clones were selected with 600 *μ*g/ml hygromycin (Invitrogen) for several weeks.

### Apoptosis assays

Three methods were used to assess PL-induced apoptotic cell death: detection of DNA fragmentation with the Cell Death Detection ELISA kit (Roche Diagnostics, Castle Hill, NSW, Australia), Western blot analysis of caspase-3 cleavage, Cyt c, AIF, or endoG release and measurement of apoptotic cells by flow cytometry (Annexin/PI, Bak activation, or activated caspase-3). The Cell Death Detection ELISA quantified the apoptotic cells by detecting the histone-associated DNA fragments (mono- and oligo-nucleosomes) generated by the apoptotic cells.^5^

### Cell fractionation and western blot analysis

Mitochondria and cytoplasm from cells were fractionated by differential centrifugation as previously described.^5^ Cytosol, mitochondria, total lysates, and immunoprecipitates were analyzed by western blot with antibody dilutions as follows: actin at 1:20 000; AIF, caspase-3, endoG, p53, Akt, p-Akt, Bid, XIAP, CoxIV at 1:2000; and Bax, Bak, Cyt c at 1:1000.

### Bak oligomerization and bak conformational change

Cells were treated with agents and incubated with 1 mM BMH in 10% DMSO or DMSO alone for 30 min at 25 °C. After centrifugation at 5000 × *g* for 25 min at 4 °C, the reaction was split into supernatant and pellet fractions. The pelleted material (10 mg total protein) was separated by SDS-PAGE and immunoblotted with anti-Bak antibody to detect Bak oligomerization.^56^ Bak conformational change was performed as described.^57^ Briefly, cells were lysed in 1% CHAPS buffer, and 250 *μ*g of protein was immunoprecipitated using anti-Bak (Ab-2), which only recognizes Bak that has undergone a conformation change. Immunoprecipitated protein was then subjected to immunoblot analysis by using anti-Bak as primary antibodies.

### Statistical analysis

Statistical analysis of the differences between the groups was performed using the Student's *t*-test with *P*<0.05 considered as statistically significant.

## Abbreviations

AIF: apoptosis-inducing factor
BMH: Bismaleimidohexane
Cyt c: cytochrome c
endoG: endonuclease G
KO: knockout
KD: knockdown
p-Akt: Akt phosphorylation
MOM: mitochondrial outer membrane
WD: wild type
