# Supplementary figures and images for: Caspase-3 feedback loop enhances Bid-induced AIF/endoG and Bak activation in Bax and p53-independent manner
Source: Cell Death Dis. 2015 Oct 15;6(10):e1919–. doi: 10.1038/cddis.2015.276 (PMC4632302; doi:10.1038/cddis.2015.276)

# Supplementary Figure 1

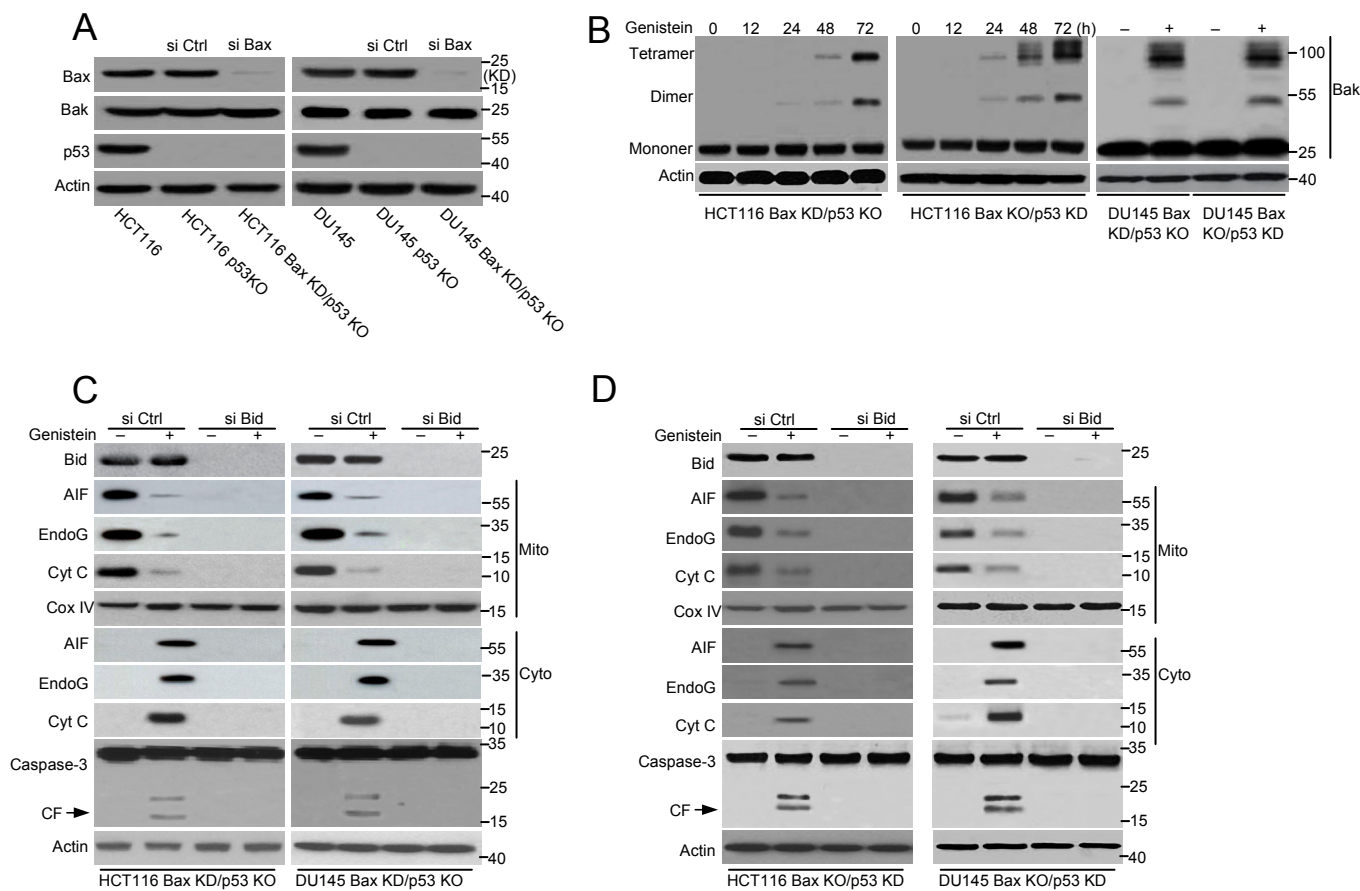

Supplement: Supplementary Figure 1 [file cddis2015276x1.pdf]
