# Supplementary material for: Caspase-3 feedback loop enhances Bid-induced AIF/endoG and Bak activation in Bax and p53-independent manner
Source: Cell Death Dis. 2015 Oct 15;6(10):e1919–. doi: 10.1038/cddis.2015.276 (PMC4632302; doi:10.1038/cddis.2015.276)
Supplement: Supplementary Figure 1 Legend [file cddis2015276x2.doc]

Supplementary Fig. 1. **A.** p53 KO cells were transiently transfected with Ctrl or Bax siRNA for 48 h and transfected cells were immunoblotted for p53, Bax, Bak detection. β-Actin was used as a protein loading control. **B.** Time-dependent analysis of Bak oligomerization in Bax KO/p53 KD and p53 KO/Bax KD cells treated with genistein (30 μM) at the indicated time. Cells were treated with genistein (30 μM), and then collected for detection. The oligomerization of Bak was assessed by cross-linking with Bismaleimidohexane (BMH) as described in Materials and methods. Treated cells were lysed in lysis buffer, and Bak was detected by Western blotting with anti-Bak antibody (Sigma, MO). β-Actin was used as a protein loading control. **C** and **D.** Bax KO/p53 KD and p53 KO/Bax KD cells were transfected with Bid and Ctrl siRNA for 48 h, and then treated with genistein for 72 h. One portion of cells was subjected to subcellular fraction to detect the release of AIF, endoG and Cyt c. the other portion of cells were collected to detect caspase activation. β-Actin and Cox IV were used as a protein loading control. All data are representative of three independent experiments.
